# Kif4A mediates resistance to neoadjuvant chemoradiotherapy in patients with advanced colorectal cancer via regulating DNA damage response: Kif4A mediates resistance to neoadjuvant chemoradiotherapy

**DOI:** 10.3724/abbs.2022068

**Published:** 2022-06-09

**Authors:** Rui Zhang, Shuanghui Liu, Bojiang Gong, Wenran Xie, Youjuan Zhao, Liang Xu, Yi Zheng, Shengnan Jin, Chunming Ding, Chang Xu, Zhixiong Dong

**Affiliations:** 1 Zhejiang Provincial Key Laboratory of Medical Genetics Key Laboratory of Laboratory Medicine Ministry of Education School of Laboratory Medicine and Life Science Wenzhou Medical University Wenzhou 325000 China; 2 Department of Colorectal Surgery the First Affiliated Hospital of Wenzhou Medical University Wenzhou 325000 China

**Keywords:** apoptosis, colorectal cancer, DNA damage response, Kif4A, neoadjuvant chemoradiotherapy

## Abstract

More and more patients with advanced colorectal cancer (CRC) have benefited from surgical resection or ablation following neoadjuvant chemoradiotherapy (nCRT), but nCRT may be ineffective and have potential risks to some patients. Therefore, it is necessary to discover effective biomarkers for predicting the nCRT efficacy in CRC patients. Chromokinesin Kif4A plays a critical role in mitosis, DNA damage repair and tumorigenesis, but its relationship with nCRT efficacy in advanced CRC remains unclear. Here, we find that Kif4A expression in pretreated tumor tissue is positively correlated with poorer tumor regression after receiving nCRT ( *P*=0.005). Knockdown of endogenous *Kif4A* causes an increased sensitivity of CRC cells to chemotherapeutic drugs 5-fluorouracil (5-FU) and Cisplatin (DDP), while overexpression of Kif4A enhances resistance of CRC cells to the chemotherapeutic drugs. Furthermore, depending on its motor domain and tail domain, Kif4A regulates DNA damage response (DDR) induced by 5-FU or DDP treatment in CRC cells. In conclusion, we demonstrate that Kif4A may be a potential independent biomarker for predicting the nCRT efficacy in advanced CRC patients, and Kif4A regulates chemosensitivity of CRC cells through controlling DDR.

## Introduction

Colorectal cancer (CRC) is the second and third most commonly diagnosed cancer in females and males, respectively [1]. Despite incidence rate of CRC gradually declined during the 2000s in the wake of widespread colonoscopy screening, it is estimated that there were 1.9 million new cases and almost 915,880 deaths from CRC in 2020 [1]. Currently, clinical patients with advanced CRC at diagnosis are usually recommended to receive systemic neoadjuvant chemoradiotherapy (nCRT) before surgery [2]. Moreover, nCRT combined with total mesenteric resection (TME) has become the standard treatment for resectable locally advanced rectal cancer (LARC) [3]. The aims of nCRT are to alleviate and control symptoms, decrease the risk of metastasis, improve quality of life, and prolong overall survival [4]. Indeed, more and more patients have benefited from nCRT. However, some patients respond poorly with little or no tumor regression, which means that nCRT may lead to overtreatment in this proportion of patients [5]. Therefore, it is necessary to find effective biomarkers for predicting the response of CRC patients to nCRT.

Chromokinesin Kif4A plays an important role in regulating multiple mitotic processes including chromosome condensation, spindle assembly and dynamics, and chromosome congression and alignment in early mitosis and spindle midzone formation, elongation and cytokinesis in late mitosis [ 6– 9] . Knockdown of *Kif4A* results in abnormal chromosome segration and aneuploidy [10]. In addition, it has been reported that Kif4A participates in DNA damage response (DDR) [ 11– 13] . Kif4A is recruited to DNA damage sites through associating with BRCA2 and then facilitates homologous recombination [11]. It has been demonstrated that Kif4A expression is closely associated with the sensitivity of lung adenocarcinoma cells to cisplatin (DDP) *in vitro* [ 12, 13] . Obviously, dysfunction of Kif4A is related to genomic instability which is intensely suspected to be a critical role in tumorigenesis. Many studies have demonstrated that Kif4A functions as an oncogene in several malignances, such as lung cancer, gastric cancer, oral cancer, hepatocellular carcinoma and CRC [ 14– 18] . Nevertheless, the relationship between Kif4A expression and clinical response of CRC patients to nCRT remains unknown.

In the present study, we detected the expression of Kif4A in pretreated biopsy specimens from patients with advanced CRC, and analyzed the relationship between Kif4A expression and nCRT response. We found that Kif4A expression is negatively correlated with nCRT response of CRC patients. Furthermore, we demonstrated that Kif4A regulates the sensitivity of CRC cells to 5-fluorouracil (5-FU) and DDP by controlling DDR.

## Materials and Methods

### Patients and the clinical database

Seventy-two patients with locally advanced CRC were recruited from the First People’s Hospital of Wenzhou Medical University (Wenzhou, China) with informed consents, as approved by the Research Ethics Committee. All patients had advanced CRC (T3–T4, N0 or T any, N1–2) and underwent nCRT from 2016 to 2020. Physical examination, carcinoembryonic antigen (CEA), routine blood test, chest enhancement CT, as well as abdomen and pelvic enhancement CT were performed before therapy. Biopsy tissues were obtained by colonoscopy from the patients before nCRT. Then, the patients were staged according to the AJCC criteria (Edition 8) [19].

### Evaluation of the nCRT efficacy

The nCRT efficacy of each patient was evaluated by Response Evaluation Criteria in Solid Tumors (RECIST) 1.1 guidelines [20] on the basis of contrast-enhanced computed tomography (CT) scans. Unidimensional measurements of target lesions and qualitative assessment of non-target disease allowed categorization of overall tumor response as complete response (CR), partial response (PR), stable disease (SD), or progressive disease (PD). The best overall response recorded from the beginning to the end of the treatment was used for further analysis.

The nCRT efficacy of patient who underwent surgery was further assessed by tumor down-staging analysis. Two pathologists blinded to clinical information analyzed the postoperative pathological results, respectively. Preoperative biopsy tissues and postoperative pathological tissues were staged according to the AJCC staging criteria to assess the tumor down-staging effect of nCRT.

### Immunohistochemical analysis of Kif4A expression

Seventy-two biopsy tumor tissue samples before nCRT were collected. The paraffin embedded specimens were sliced in 5 μm of thickness, and Kif4A expression level was detected by immunohistochemistry (IHC). Tissue sections were dewaxed by xylene, and hydrated by a graded ethanol series and distilled water. After antigen retrieval, 5% goat serum was added (Beyotime, Shanghai, China) for blocking at room temperature for 30 min. Then, rabbit anti-Kif4A antibodies at 1:200 were used to stain the samples at 4°C for 12 h. HRP-conjugated goat anti-rabbit secondary antibodies were added and incubated at room temperature for 30 min. Signal was revealed using 3,3′-diaminobenzidine (3,3′-DAB), and hematoxylin was used for counterstaining. Finally, the slices were dehydrated by a graded ethanol series and xylene, mounted and examined by microscopy. The images were analyzed by IOD (integrated option density) using Image-Pro Plus 6.0 software.

### Plasmids, siRNAs and antibodies

Mammalian expression plasmids of Kif4A and its mutants were generated as described previously [10]. All constructs were fully sequenced. Nonsense control siRNA (siNC: 5′-TTCTCCGAACGTGTCACGTTT-3′) and siRNAs specific targeting to 3′-UTR (siKif4A: 5′-GGAATGAGGTTGTGATCTT-3′) or CDS region (siKif4A-CDS: 5′-GGAATGAGGTTGTGATCTTTT-3′) of Kif4A were synthesized by Genepharma (Shanghai, China).

Polyclonal rabbit anti-Kif4A were generated as previously described [10]. Mouse anti-GAPDH antibody (60004-1-Ig) was purchased from Proteintech (Rosemont, USA), mouse anti-γH2AX antibody (05-636) and mouse anti-GFP antibody (G6539) were purchased from Merk Millipore (Billerica, USA). All secondary antibodies were obtained from Life Technologies (Waltham, USA).

### Cell culture, transfection and drug treatment

CCD 841 CoN cells were provided by Dr Haishan Huang of Wenzhou Medical University (Wenzhou, China). All CRC cell lines were purchased from the American Type Culture Collection (ATCC; Manassas, USA) where these cell lines were characterized by DNA fingerprinting and isozyme detection. CCD 841 CoN, SW480, SW620 and LoVo cells are cultured in RPMI 1640 medium (Gibco, Carlsbad, USA) supplemented with 10% fetal bovine serum (Gibco). HCT116 and HT-29 cells were cultured in DMEM medium (Gibco) supplemented with 10% fetal bovine serum. Caco-2 cells were cultured in MEM medium (Gibco) supplemented with 20% fetal bovine serum. All cells were cultured at 37°C in 5% CO _2_. 5-FU (Merk Millipore) or DDP (Solarbio, Shanghai, China) treatment consisted of incubation with drugs at indicated concentration for indicated time. Plasmids and siRNAs transfection were conducted using Lipofectamine 3000 (Life Technologies) according to the manufacturer’s protocol.

### Western blot and immunofluorescence analysis

Cells were harvested or fixed for western blot or immunofluorescence analysis as previously described [21]. In brief, for western blot analysis, cells were harvested and lysed in RIPA buffer. Cell lysates with equal amount of total protein were subject to SDS-PAGE, transferred to PVDF membrane and then immunoblotted with corresponding antibodies. For immunofluorescence analysis, cells grown on glass coverslips were fixed and immunostained with indicated antibodies. Cells were photographed using a NIKON A1 confocal microscope (Nikon, Tokyo, Japan).

### Quantitative real-time PCR (qRT-PCR)

Total RNA was isolated using the Trizol reagent (Invitrogen, Carlsbad, USA) according to manufacturer’s instructions. One microgram of RNA was used for cDNA synthesis using a Reverse Transcriptase Reaction kit (Promega, Madison, USA). qPCR was performed on a Bio-Rad CFX 96 Touch System (Bio-Rad, Hercules, USA) using SYBR Green (Tiangen, Beijing, China) as a dsDNA-specific fluorescent dye. *GAPDH* was used for standardizing *Kif4A* mRNA level. Amplification primers were forward: 5′-CTGCAATTGGTTGGCGTCTC-3′ and reverse: 5′-CAGCGCCACTCTTACAGGAA-3′ for *Kif4A* and forward: 5′-TTCATTGACCTCAACTACATGGTTTAC-3′ and reverse: 5′-TGACAAGCTTCCCGTCTCA-3′ for *GAPDH*. The mRNA levels were calculated using the 2 ^–ΔΔCt^ method [22].

### Chemotherapeutic drug sensitivity assay

CCK8 assay was carried out to determine the sensitivity of different cells to 5-FU or DDP treatment. Briefly, cells were plated in 96-well plates (3000 cells/well) and transfected with Kif4A siRNA or indicated plasmids for 24 h. Cells were treated with 5-FU or DDP at indicated doses for indicated time intervals, and CCK8 solution (Dojindo, Kyushu, Japan) was added after treatment. Cell viability was determined by measuring the absorbance at 450 nm.

### Cell apoptosis assay

After transfection or drug treatment, cells were collected, and then stained using the Annexin V/7-AAD Apoptosis Detection kit (KeyGen Biotech, Nanjing, China) according to the manufacturer’s protocol. Data was acquired using a BD FACSVerse system and BD FACSuite software (BD Biosciences, Franklin Lakes, USA).

### Statistical analysis

All of the statistical analysis was performed using the SPSS 20.0 statistical software package (SPSS Inc., Chicago, USA). The Pearson chi-square test was used to examine the correlation between Kif4A expression and clinicopathological characteristics. Student’s *t*-test was used to detect the relationship between Kif4A and tumor TNM down-staging and calculate the statistical significance of the experimental data. Data are expressed as the mean±standard deviation (SD) form 3 independent experiments. *P*<0.05 is considered statistically significant.

## Results

### Kif4A expression in pretreated biopsy specimens is negatively correlated with the nCRT response in patients with advanced CRC

**Figure 1:**
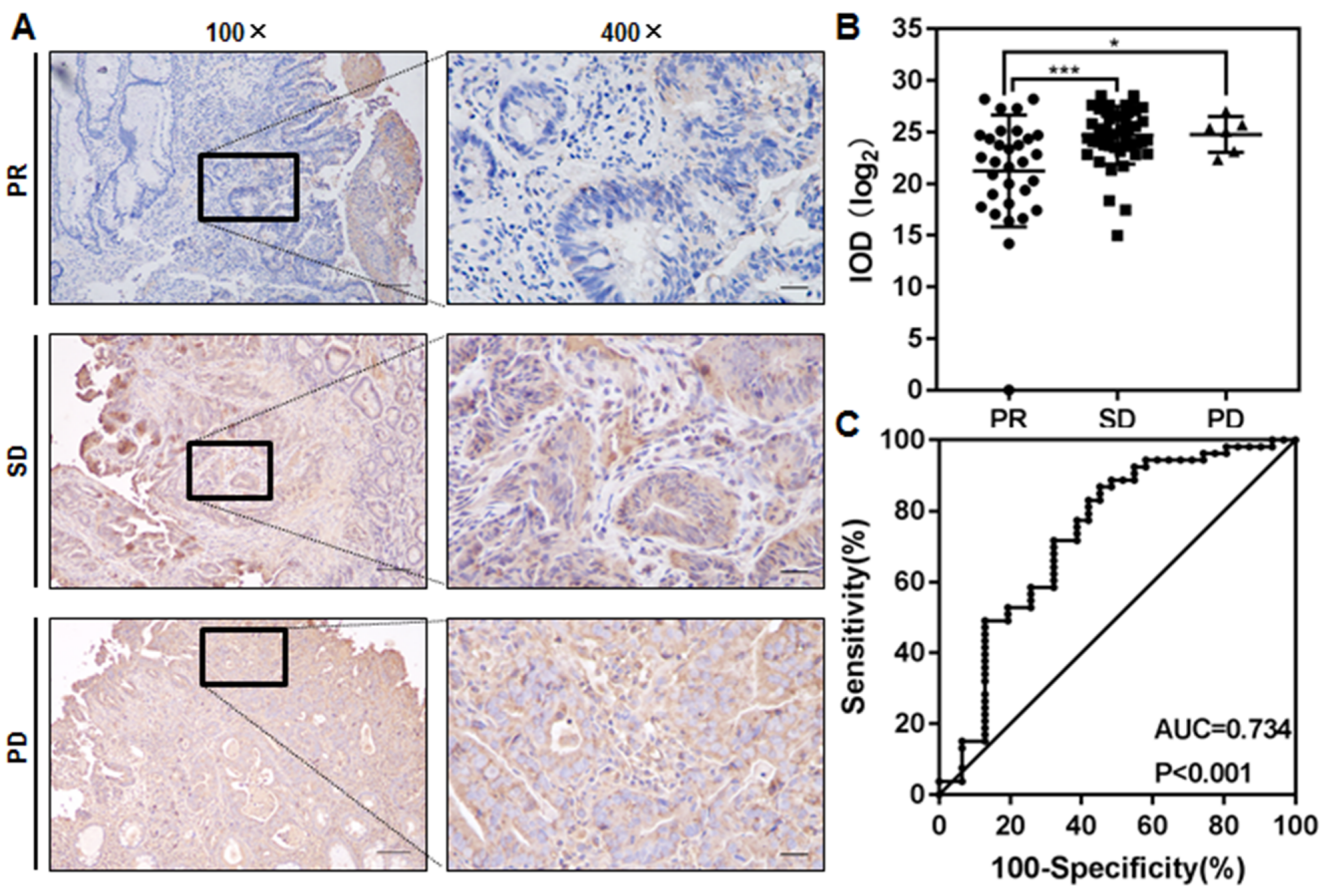
Figure 1 Kif4A expression is negatively correlated with the nCRT response of the patients with advanced CRC (A) IHC analysis the expression of Kif4A in pretreated biopsy specimens from patients with advanced CRC. Left panel, magnification 100×, scale bar: 100 μm; right panel, magnification 400×, scale bar: 30 μm. PR: partial response; SD: stable disease; PD: progressive disease. (B) Kif4A expression in biopsy specimens was quantified by calculating the IOD of each stained area (IOD/area) and its relationship with the nCRT response was analyzed (* P<0.05, and *** P<0.001). (C) ROC analysis for discriminating PR patients ( n=25) and SD/PD patients ( n=47) with an AUC value of 0.734.

In order to explore the relationship between Kif4A expression and the nCRT efficacy, we collected pretreated biopsy specimens from seventy-two patients prior to nCRT, and the clinicopathological characteristics of these patients were summarized in Table 1. Kif4A expressions in tumor tissues were detected by IHC and quantified by calculating the IOD of each stained area (IOD/area). The nCRT efficacy was assessed by RECIST 1.1 based on the clinical images before and after nCRT, and the patients were divided into PR (30/72), SD (36/72) and PD (6/72) according to the assessment system [20]. It was found that Kif4A expression in tumor tissues of group PR was significantly lower than those in groups SD and PD ( Figure 1A,B).

**Table:** **Table 1** The clinicopathological characteristics of the patients

Variable	Patients
Gender	
Male	54 (75.0%)
Female	18 (25.0%)
Age	
≤ 60	36 (50.0%)
>60	36 (50.0%)
Course (period) of treatment	
≥ 4	20 (27.8%)
<4	52 (72.2%)
Distance to anal verge, cm ( *n*=70)	
≤ 5	26 (37.1%)
>5	44 (62.9%)
CEA, ng/mL	
≤ 5	28 (38.9%)
>5	44 (61.1%)
CA19-9, U/mL	
≤ 37	53 (73.6%)
>37	19 (26.4%)
AFP3, mg/mL ( *n*=71)	
≤ 25	70 (98.6%)
>25	1 (1.4%)
Treatment group	
5-FU alone	32 (44.4%)
Oxaliplatin alone	17 (23.6%)
5-FU plus Oxaliplatin	23 (31.9%)
cT stage ( *n*=62)	
cT1–cT2	5 (8.1%)
cT3–cT4	57 (91.9%)
cN stage ( *n*=62)	
cN0	7 (11.3%)
cN1–cN2	55 (88.7%)
cTNM stage ( *n*=62)	
II	5 (8.1%)
III	53 (85.5%)
IV	4 (6.4%)
EGFR ( *n*=34)	
Positive	21 (61.8%)
Negative	13 (38.2%)
Her-2 ( *n*=34)	
Positive	4 (11.8%)
Negative	30 (88.2%)

The area under the curve (AUC) value for the receiver operating characteristic (ROC) curve distinguishing patients with partial remission and non-remission (SD and PD) was 0.734 [95% confidence interval (CI), 0.6155 to 0.8525; *P*<0.001], and the maximum log likelihood estimate value was 25.17 ( Figure 1C). Based on this definition, Kif4A expression was low (log _2_(IOD)<25) in 66.7% (48/72) and high [log _2_(IOD)>25] in 33.3% (24/72) of these patients ( Table 2). We analyzed the association of Kif4A expression with clinicopathological parameters of these patients, and found that Kif4A expression was significantly correlated with cT staging ( *P*=0.006). In contrast, no striking correlations between Kif4A expression and gender, age, treatment period, anal distance, CEA, cN, cTNM, and EGFR or HER-2 expression in pathological tissue were observed ( *P*>0.05; Table 2).

**Table:** **Table 2** Correlation of Kif4A expression with clinicopathological factors

Variable	Patient	Kif4A expression	*P* value
		low level (Log _2_IOD<25)	high level (Log _2_IOD>25)	
Gender				0.24
Male	54 (75.0%)	38 (52.8%)	16 (22.2%)	
Female	18 (25.0%)	10 (13.9%)	8 (11.1%)	
Age				0.61
≤ 60	36 (50.0%)	23 (31.9%)	13 (18.1%)	
>60	36 (50.0%)	25 (34.7%)	11 (15.3%)	
Course (period) of treatment				0.19
≥ 4	20 (27.8%)	11 (15.3%)	9 (12.5%)	
<4	52 (72.2%)	37 (51.4%)	15 (20.8%)	
Distance to anal verge, cm ( *n*=70)				0.37
≤ 5	26 (37.1%)	15 (21.4%)	11 (15.7%)	
>5	44 (62.9%)	30 (42.9%)	14 (20.0%)	
CEA, ng/mL				0.49
≤ 5	28 (38.9%)	20 (27.8%)	8 (11.1%)	
>5	44 (61.1%)	28 (38.9%)	16 (22.2%)	
CA19-9, U/mL				0.34
≤ 37	53 (73.6%)	37 (51.4%)	16 (22.2%)	
>37	19 (26.4%)	11 (15.3%)	8 (11.1%)	
cT stage ( *n*=62)				0.006
cT1–cT2	5 (8.5%)	0 (0.0%)	5 (8.5%)	
cT3–cT4	57 (91.5%)	39 (62.9%)	18 (29.0%)	
cN stage ( *n*=62)				1.00
cN0	7 (11.3%)	4 (6.5%)	3 (4.8%)	
cN1–cN2	55 (88.7%)	35 (56.5%)	20 (32.3%)	
cTNM stage ( *n*=62)				0.29
II	5 (8.1%)	4 (6.5%)	1 (1.6%)	
III	53 (85.5%)	34 (54.8%)	19 (30.6%)	
IV	4 (6.5%)	1 (1.6%)	3 (4.8%)	
EGFR ( *n*=34)				0.21
Positive	21 (61.8%)	18 (52.9%)	3 (8.8%)	
Negative	13 (38.2%)	8 (23.5%)	5 (14.7%)	
Her-2 ( *n*=34)				0.55
Positive	4 (11.8%)	4 (11.8%)	0 (0.0%)	
Negative	30 (88.2%)	22 (64.7%)	8 (23.5%)	

We further analyzed the relationship between Kif4A expression and the nCRT efficacy in these patients, and the results showed that Kif4A expression was negatively correlated with the nCRT efficacy of the patients ( *P*=0.005; Table 3). Meanwhile, consistent with previous reports, we also found that the expression levels of CEA and HER-2 were significantly correlated with the therapeutic response to nCRT in patients with advanced CRC ( *P*<0.05; Table 3) [ 23, 24] .

**Table:** **Table 3** Correlation of different clinicopathological characteristics with the nCRT efficacy

Variable	Patient	nCRT Efficacy	*P* value
		PR	SD and PD	
Gender				0.19
Male	54 (75.0%)	21 (29.2%)	33 (45.8%)	
Female	18 (25.0%)	4 (5.6%)	14 (19.4%)	
Age				0.45
≤ 60	36 (50.0%)	14 (19.4%)	22 (30.6%)	
>60	36 (50.0%)	11 (15.3%)	25 (34.7%)	
Course [period] of treatment				0.09
≥ 4	20 (27.8%)	10 (13.9%)	10 (13.9%)	
<4	52 (72.2%)	15 (20.8%)	37 (51.4%)	
Distance to anal verge, cm ( *n*=70)				0.43
≤ 5	26 (37.1%)	11 (15.7%)	15 (21.4%)	
>5	44 (62.9%)	14 (20.0%)	30 (42.9%)	
CEA, ng/mL				0.007
≤ 5	28 (38.9%)	15 (20.8%)	13 (18.1%)	
>5	44 (61.1%)	10 (13.9%)	34 (47.2%)	
CA19-9, U/mL				0.14
≤ 37	53 (73.6%)	21 (29.2%)	32 (44.4%)	
>37	19 (26.4%)	4 (5.6%)	15 (20.8%)	
cT stage ( *n*=62)				0.64
cT1–cT2	5 (8.1%)	1 (1.6%)	4 (6.5%)	
cT3–cT4	57 (91.9%)	23 (37.1%)	34 (54.8%)	
cN stage ( *n*=62)				0.69
cN0	7 (11.3%)	2 (3.2%)	5 (8.1%)	
cN1–cN2	55 (88.7%)	22 (35.5%)	33 (53.2%)	
cTNM stage ( *n*=62)				1.00
II	5 (8.1%)	2 (3.2%)	3 (4.8%)	
III	53 (85.5%)	21 (33.9%)	32 (51.6%)	
IV	4 ( 6.5%)	1 (1.6%)	3 (4.8%)	
EGFR ( *n*=34)				0.72
Positive	21 (61.8%)	8 (23.5%)	13 (38.2%)	
Negative	13 (38.2%)	6 (17.6%)	7 (20.6%)	
Her-2 ( *n*=34)				0.02
Positive	4 (11.8%)	4 (11.8%)	0 (0.0%)	
Negative	30 (88.2%)	10 (29.4%)	20 (58.8%)	
Kif4A expression				0.005
Positive	24 (33.3%)	3 (4.2%)	21 (29.2%)	
Negative	48 (66.7%)	22 (30.6%)	26 (36.1%)	

Pathologic down-stage was also used to evaluate the nCRT response. We investigated the relationship between Kif4A expression and tumor down-staging, and the results showed that there was no striking association of Kif4A expression with down-staging ( Supplementary Figure S1A–C and Supplementary Table S1). Taken together, chromokinesin Kif4A expression displays a negative correlation with the nCRT response in patients with advanced CRC.

### Kif4A expression is closely correlated with the sensitivity of CRC cells to chemotherapeutic drug treatment

**Figure 2:**
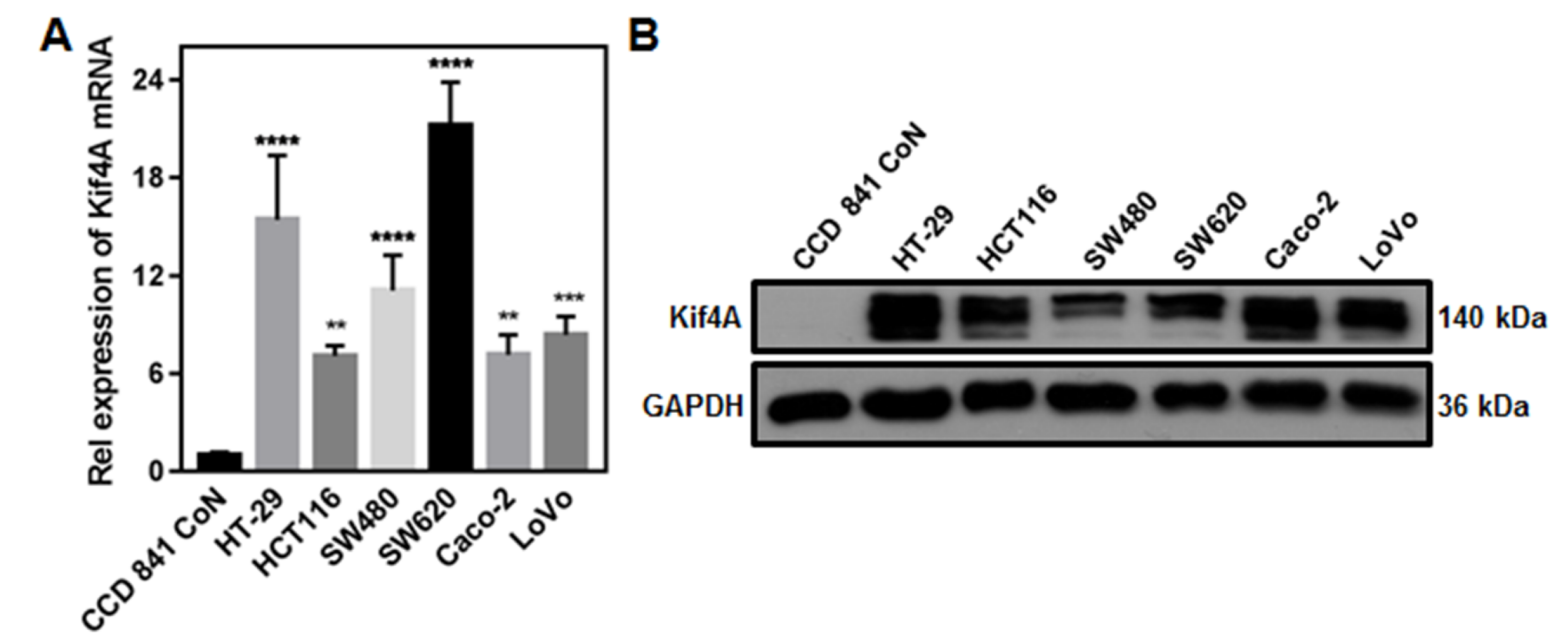
Figure 2 Kif4A is upregulated in CRC cells Normal colon epithelial cell (CCD 841 CoN) and different CRC cells were harvested to detect the expression levels of Kif4A mRNA (A) and protein (B). Data are presented as the mean±SD derived from three independent experiments. ** P<0.01, *** P<0.001 and **** P<0.0001 compared to CCD 841 CoN cells.

To investigate the mechanism of Kif4A in regulating the nCRT efficacy, we detected Kif4A expression in different CRC cells at both mRNA and protein levels. As shown in Figure 2, compared with those in the normal colon epithelial cell (CCD 841 CoN), the mRNA and protein levels of Kif4A were significantly upregulated in all of detected CRC cells, including HT-29, HCT116, SW480, SW620, Caco-2 and LoVo cells ( Figure 2).

**Figure 3:**
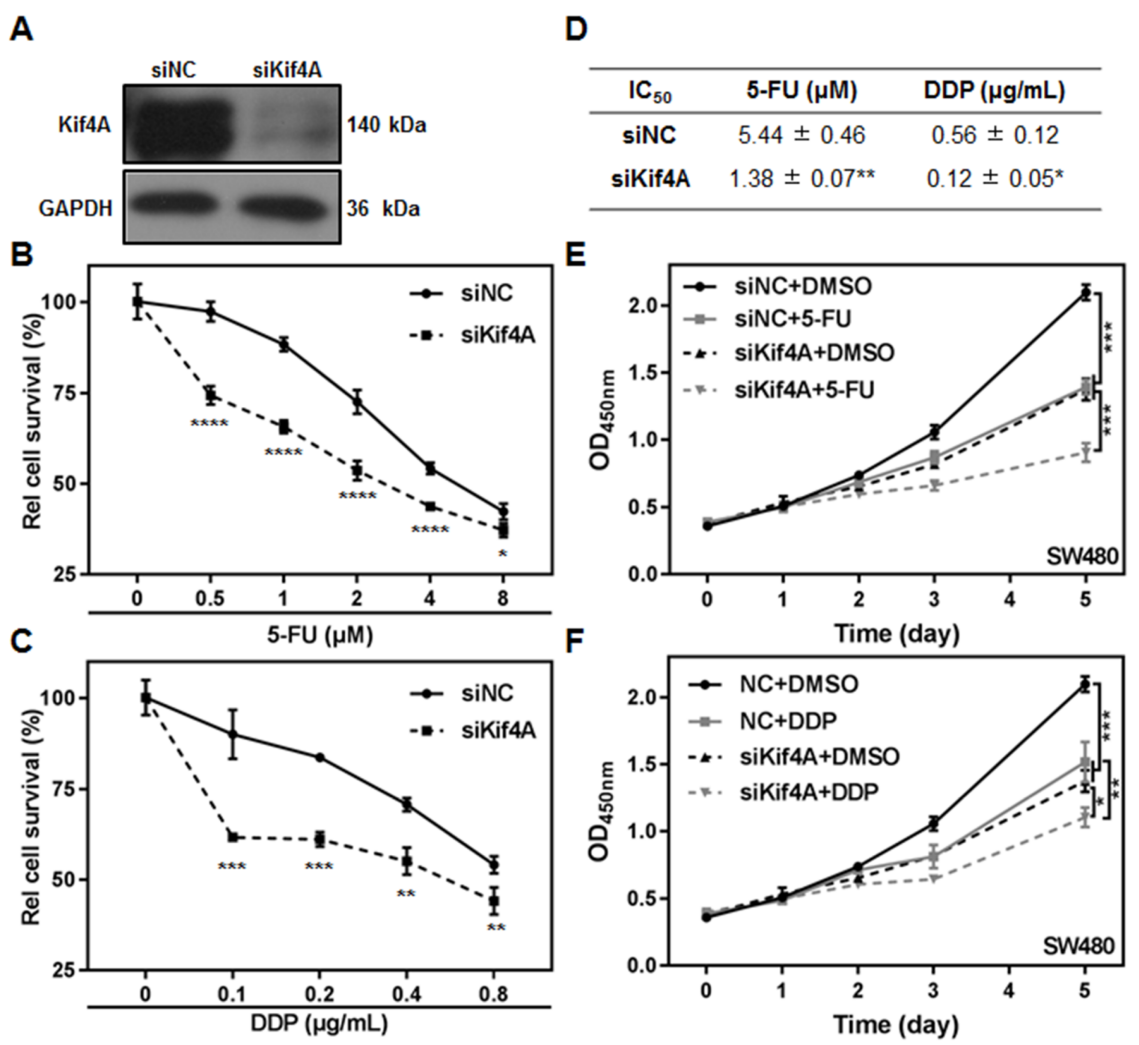
Figure 3 Depletion of Kif4A enhances sensitivity of SW480 cells to 5-FU or DDP treatment (A) The expression of Kif4A in SW480 cells after transfection with indicated siRNA was detected by western blot analysis. (B,C) SW480 cells were seeded on 96 well plate and transfected with indicated siRNAs. Twenty-four hours later, the cells were treated with 5-FU (B) or DDP (C) at indicated doses for 48 h. The cells survival was detected by CCK8 assay. (D) The effect of Kif4A depletion on IC 50 of SW480 cells to 5-FU or DDP was evaluated. Data are presented as the mean± SD (standard deviation) derived from three independent experiments. (E-F) SW480 cells were transfected with indicated siRNA and treated with DMSO, 5-FU (E) or DDP (F), cells survival was detected at indicated times using CCK8 kit. Data are presented as the mean±SD derived from three independent experiments. * P<0.05, ** P<0.01, *** P<0.001, and **** P<0.0001.

We further investigated the effect of Kif4A expression on the sensitivity of CRC cells to chemotherapeutic drugs *in vitro*. SW480 cells were transfected with siRNA targeting the Kif4A 3′-UTR (siKif4A) or nonsense control siRNA (siNC), and then were treated with different doses of 5-FU or DDP. Western blot analysis results showed that Kif4A siRNA effectively inhibited the endogenous Kif4A expression ( Figure 3A). Compared with the control cells, depletion of Kif4A strikingly increased the sensitivity of CRC cells to both 5-FU and DDP, and resulted in a significant decrease in the IC _50_ values ( Figure 3B–D). Meanwhile, we detected the proliferation of Kif4A-depleted cells and control cells at different time points after 5-FU or DDP treatment. The results showed that, although Kif4A depletion or drugs treatment alone could inhibit cell proliferation in a time-dependent manner, the combination of Kif4A depletion and drug treatment collaboratively prevented the cell proliferation ( Figure 3E,F). We also examined the effects of Kif4A deletion on chemotherapeutic drugs sensitivity of other CRC cells. The results showed that Kif4A knockdown also significantly enhanced the sensitivity of SW620 cells to 5-FU or DDP ( Supplementary Figure S2A–D). In addition, to eliminate the potential possibility of off-target using a single siRNA, we synthesized another siRNA targeting Kif4A CDS region (siKif4A-CDS), and evaluated the effect of Kif4A knockdown by siRNA on the chemosensitivities of CRC cells. It was found that siKif4A-CDS transfection also dramatically inhibited the expression of Kif4A in SW480 cells and enhanced the chemosensitivity of the cells to both 5-FU and DDP treatment ( Supplementary Figure S2E–G). In contrast, overexpression of Kif4A caused a significantly increased the resistance of SW480 cells to DDP treatment ( Supplementary Figure S3A,B). These results demonstrate that the expression level of Kif4A is negatively correlated with chemotherapeutic sensitivity of CRC cells *in vitro*.

### Kif4A depletion enhances cell apoptosis induced by 5-FU or DDP treatment

**Figure 4:**
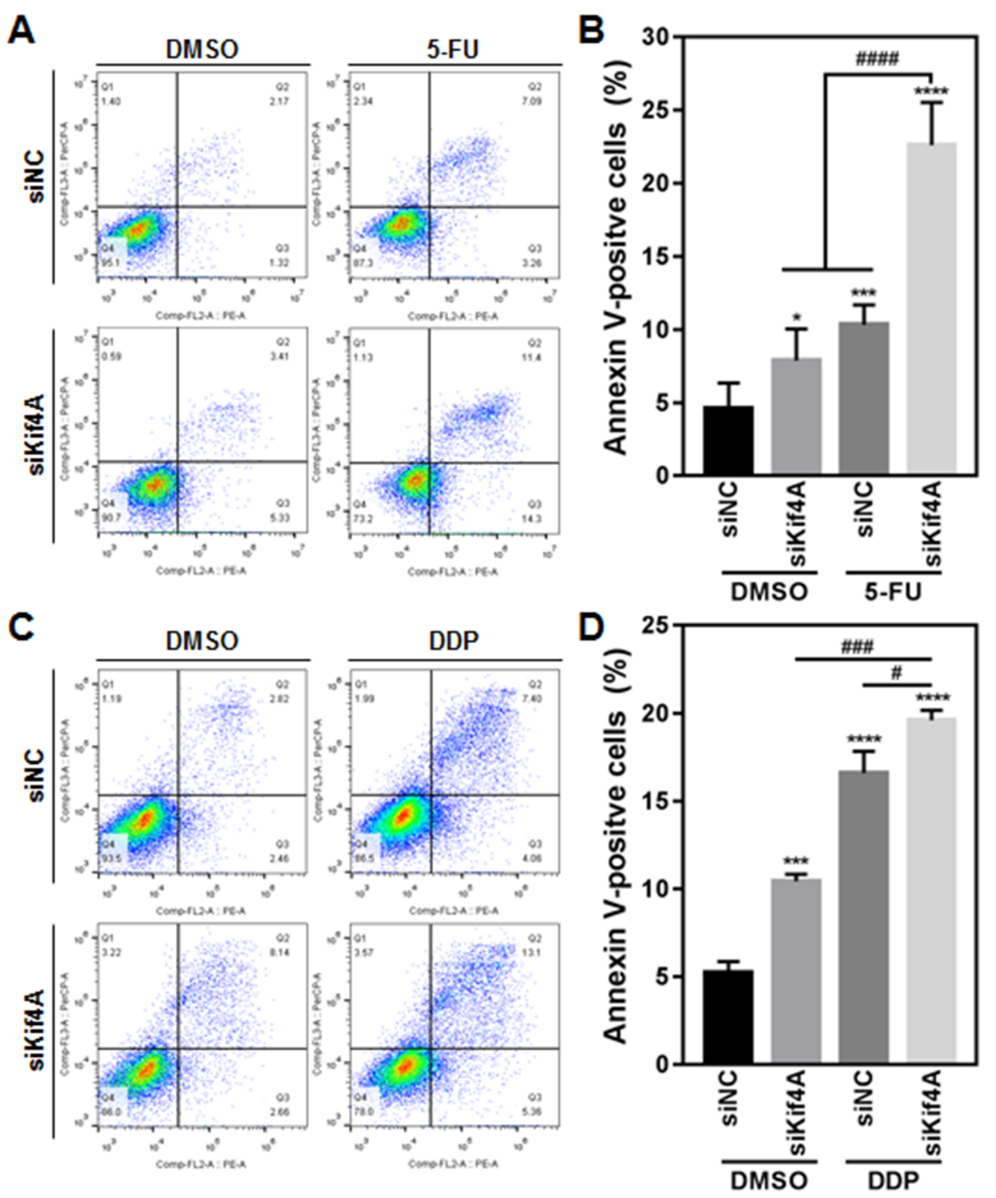
Figure 4 Kif4A regulates cell apoptosis of CRC cells in response to 5-FU or DDP treatment SW480 cells were transfected with indicated siRNA for 24 h, and then treated with 5-FU (A,B) or DDP (C,D) for 48 h. The cell apoptosis was assessed by Annexin V/7-AAD staining and analyzed by FACS. The percentage of apoptotic cells (Annexin V-positive cells) was counted. Data are shown as the mean±SD from three independent experiments. * P<0.05, *** P<0.001, **** P<0.0001 vs siNC treated with DMSO; # P<0.05, ### P<0.001 and #### P<0.0001 vs indicated treatment.

We further examined the difference in chemotherapy-induced apoptosis between Kif4A-depleted cells and control cells. Kif4A-depleted SW480 cells and control cells were treated with 5-FU or DDP, and cell apoptosis was analyzed by Annexin V/7-AAD assay. The results showed that Kif4A depletion or drug treatment alone could induce an accumulation of Annexin V-positive cells, while depletion of Kif4A caused a significant increase of Annexin V-positive cells after 5-FU ( Figure 4A,B) or DDP ( Figure 4C,D) treatment. Therefore, Kif4A participates in regulating apoptosis induced by chemotherapeutic drug treatment.

### Kif4A affects the chemotherapeutic sensitivity by regulating DDR induced by chemotherapeutic drugs

**Figure 5:**
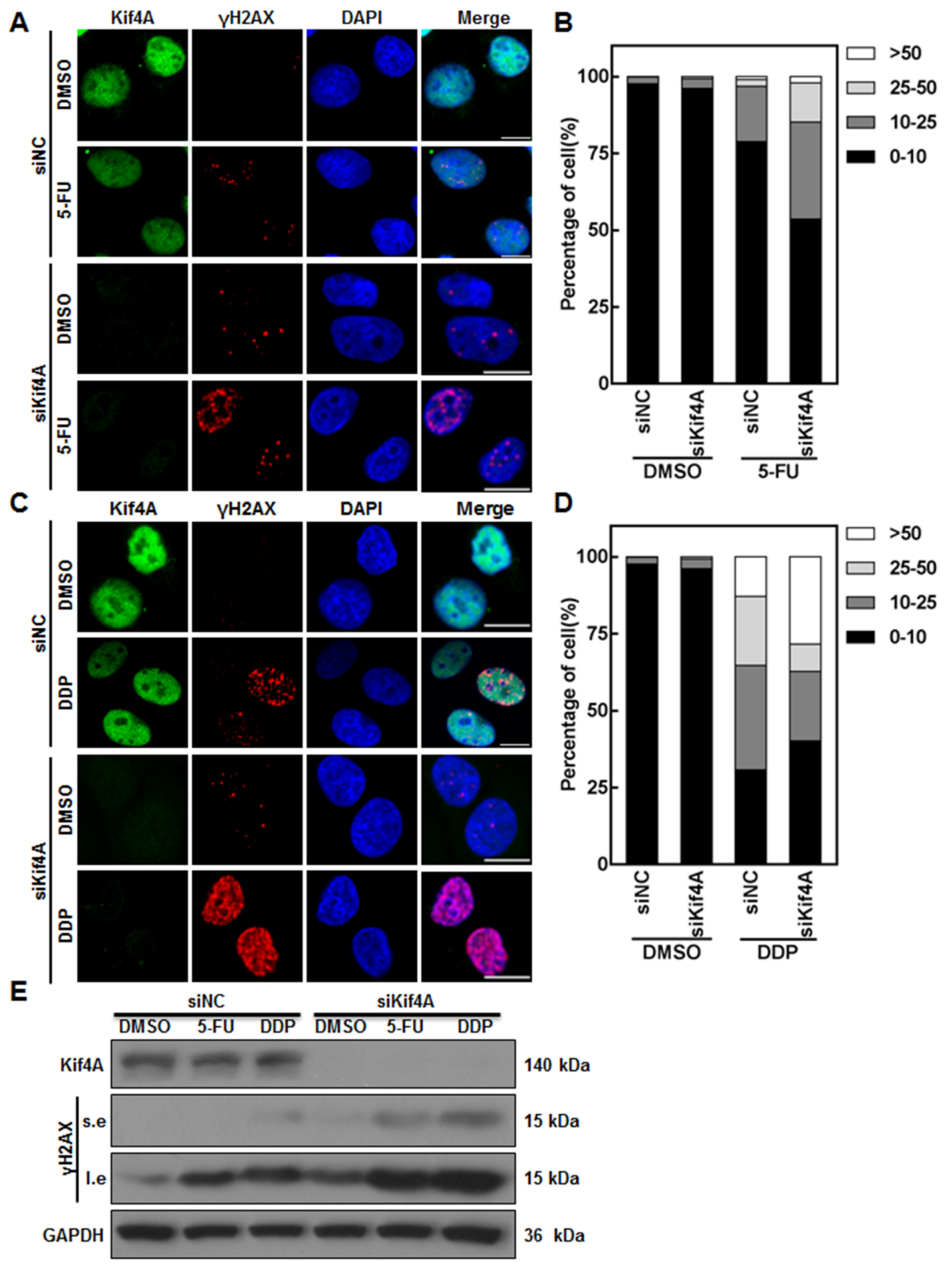
Figure 5 Depletion of Kif4A causes an increase of DDR in SW480 cells in response to 5-FU or DDP treatment (A–D) SW480 cells were transfected with indicated siRNAs for 24 h, and then treated with 5-FU or DDP for 48 h. The cells were fixed and stained with anti-Kif4A antibodies and γH2AX antibody. The cell nuclei were stained with DAPI. Scale bar: 5 μm. (B) and (D) show the percentage of cells with different number of γH2AX foci calculated from (A) and (C), respectively, and at least 50 cells from three independent experiments were counted. (E) SW480 cells were transfected with indicated siRNAs for 24 h, and then treated with 5-FU or DDP for 48 h. The cells were harvested, lysed, and subject to western blot analysis using indicated antibodies. s.e.: short exposure; l.e.: long exposure.

The primary mechanism by which chemotherapeutic drugs 5-FU and DDP kill tumor cells is interrupting DNA replication and inducing DNA damage, which further activate DNA damage checkpoint and ultimately cause cell death [25]. It has been reported that Kif4A participates in DNA damage repair [ 11– 13] . Therefore, we speculate that the role of Kif4A in controlling chemotherapeutic sensitivity may be attributed to its function in regulating DDR. To test this, we detected the DNA damage signals in control and Kif4A-depleted cells after 5-FU or DDP treatment. Immunostaining results showed that, compared with controls, Kif4A depletion caused a few γH2AX (phosphorylated H2AX) foci formation, further indicating that Kif4A is involved in regulating DDR. After 5-FU or DDP treatment, Kif4A-depleted cells displayed a striking increase of γH2AX foci compared with controls ( Figure 5A–D and Supplementary Figure S4A–D). Consistently, western blot analysis also showed that, after 5-FU or DDP treatment, the γH2AX level in Kif4A-depleted cells were significantly higher than that in controls ( Figure 5E). In contrast, compared with GFP-expressing cells, γH2AX foci and γH2AX level in Kif4A-overexpressing cells were remarkably decreased after 5-FU or DDP treatment ( Supplementary Figure S5A–E). These results indicate that Kif4A negatively regulates DDR induced by chemotherapeutic drugs in CRC cells.

### Kif4A regulates DDR of CRC cells depending on its motor and tail domains

**Figure 6:**
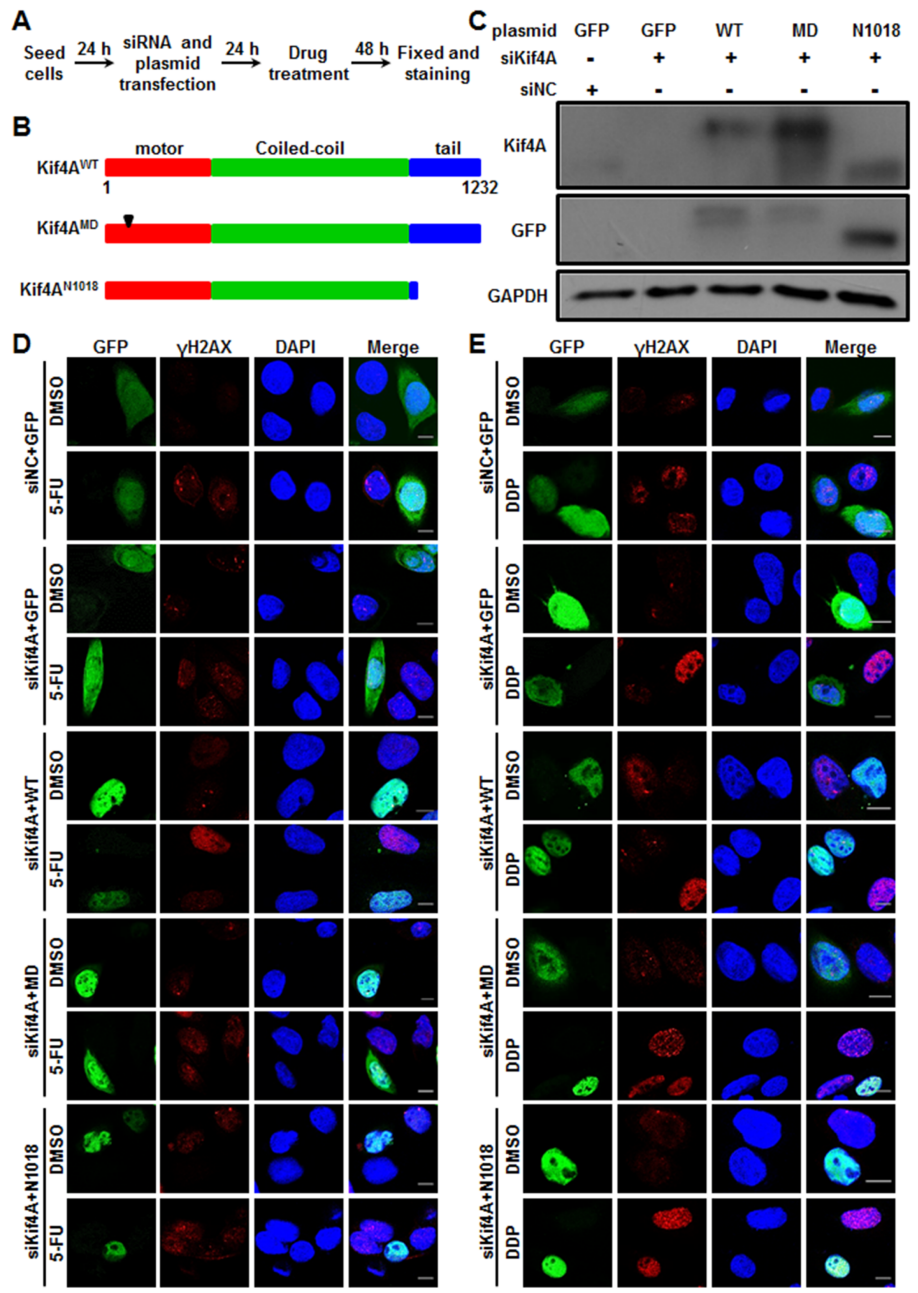
Figure 6 Kif4A regulates chemotherapeutic sensitivity and DDR of SW480 cells depending on its motor and tail domains (A) Experimental outline of the Kif4A-RNAi rescue experiments. (B) Schematic representation of the protein domain of Kif4A and its mutants. (C) SW480 cells were transfected with siNC or siKif4A together with plasmids of pEGFP, pEGFP-Kif4A WT, pEGFP-Kif4A MD or pEGFP-Kif4A N1018 for 48 h. The cells were lysed and subject to western blot analysis using indicated antibodies. (D-E) The transfected cells following (A) were treated with 5-FU (D) or DDP (E) for 48 h, and then fixed and stained with anti-γH2AX antibody and DAPI. Scale bar: 5 μm.

It has been reported that Kif4A regulates DDR by interacting with BRCA2 through its tail domain [11]. Our previous study revealed that Kif4A regulates DDP resistance by interacting with LRP (lung resistance-related protein) and then transporting LRP-based vaults, which are depend on Kif4A tail domain and motor domain, respectively [13]. Therefore, we sought to investigate which domain(s) of Kif4A is (are) critical for regulating chemotherapeutic sensitivity of CRC cells. To this end, we performed knockdown-rescue assay following the outlined protocol ( Figure 6A). SW480 cells were transfected with Kif4A siRNA targeting the 3′-UTR of *Kif4A* mRNA together with vectors expressing GFP (control), GFP-Kif4A ^WT^, GFP-Kif4A ^MD^ (motor dead), or GFP-Kif4A ^N1018^ (lacking the tail domain) by using cDNAs lacking the native 3′-UTR ( Figure 6A,B). Western blot analysis showed that Kif4A siRNA could effectively ablate the expression of endogenous Kif4A protein but did not affect ectopic expression of GFP-Kif4A ^WT^, GFP-Kif4A ^MD^ or GFP-Kif4A ^N1018^ protein ( Figure 6C). Immunostaining of γH2AX revealed that, after 5-FU or DDP treatment, cells expressing GFP-Kif4A ^WT^ could inhibit γH2AX foci formation on account of lacking endogenous Kif4A ( Figure 6D,E and Supplementary Figure S6A,B). However, like cells with Kif4A depletion, cells expressing GFP-Kif4A ^MD^ or GFP-Kif4A ^N1018^ protein but lacking endogenous Kif4A displayed similar γH2AX foci formation after 5-FU or DDP treatment ( Figure 6D,E and Supplementary Figure S6A,B). Therefore, Kif4A regulates DDR induced by chemotherapeutic drugs in CRC cells depending on its motor domain and tail domain.

## Discussion

In this study, we found that Kif4A expression in biopsy samples of patients with advanced CRC is negatively correlated with the chemosensitivity of patients to nCRT. Kif4A controls the sensitivity of CRC cells to 5-FU or DDP by regulating DDR, which is dependent on its motor domain and tail domain. Our results indicate that chromokinesin Kif4A may be used as a prospective biomarker to predict the nCRT efficacy in advanced CRC patients.

Considering the unsatisfactory therapeutic response to nCRT in some patients with advanced CRC, it is clinically relevant to identify in advance the patients who can benefit from nCRT. Several biomarkers have been investigated and categorized as prognostic and predictive biomarkers, such as p53, EGFR and CEA [26]. However, most of these biomarkers are restricted for clinical application because the conclusions from different studies on the association of these markers with nCRT response are controversial. For example, p53 is a critical protein which regulates cell cycle, DNA damage response and cell death [27]. Some studies found that absence of p53 in biopsy specimens is closely correlated with good response to nCRT [ 28, 29] . Conversely, several other studies did not find any association between p53 expression and nCRT response [26]. Therefore, a prospective biomarker for nCRT response prediction needs to be evaluated in larger trials using effective assessment index. Our data showed that chromokinesin Kif4A could be used to differentiate CRC patients who have different response to nCRT. However, the specificity and sensitivity were not very satisfactory when using Kif4A as a single biomarker to predict nCRT efficacy. Therefore, it is necessary to further investigate the efficiency of multivariate analysis combining Kif4A with other CRC biomarkers, such as JMJD2D, ARRB2 and HER2 [ 23, 30, 31] .

Kif4A plays a critical role in mitotic progression by regulating chromosome condensation, congression, and cytokinesis [ 32, 33] . However, Kif4A localizes in the cytoplasm and nucleus, and regulates multiple cellular functions at the interphase [ 34, 35] . In addition, Hou *et al*. [14] found that Kif4A is upregulated in CRC tumor tissue and cells, and regulates cell proliferation by inhibiting p21 transcription. Here, we demonstrated that Kif4A expression is negatively correlated with nCRT response in advanced CRC patients, and Kif4A regulates the sensitivity of CRC cells to 5-FU or DDP by regulating DDR. Wu *et al*. [11] reported that Kif4A participates in regulating DDR by interacting with BRCA2 through its tail domain, and our previous study also found that Kif4A regulates DDP resistance of A549 cells by interacting with and transporting LRP-based vaults [13]. In this study, we found that both the motor domain and the tail domain are required for Kif4A-mediated DDR regulation in CRC cells.

In conclusion, we demonstrated that Kif4A expression in pretreated biopsy specimens is negatively correlated with nCRT response in patients with advanced CRC and that Kif4A regulates the sensitivity of CRC cells to chemotherapeutic drugs by regulating DDR. Our data indicate that Kif4A may be a prospective marker for nCRT efficacy prediction in the patients with advanced CRC before nCRT treatment.

## Supplementary Data

Supplementary data is available at *Acta Biochimica et Biophysica Sinica* online.
